# Antoni van Leeuwenhoek 1723–2023: a review to commemorate Van Leeuwenhoek’s death, 300 years ago: For submission to Antonie van Leeuwenhoek journal of microbiology

**DOI:** 10.1007/s10482-023-01859-4

**Published:** 2023-07-31

**Authors:** Lesley A. Robertson

**Affiliations:** Department of Biotechnology and Science Centre, Delft University of Technology, Van der Burghweg 1, 2628 CS Delft, The Netherlands

**Keywords:** Leeuwenhoek, Methods, Modern equipment, Micro-photography, Simple microscopes

## Abstract

In the 300 years since Van Leeuwenhoek died, some of the details around his life and his work have provided material for discussion or dispute. As archives and libraries are being scanned and technology improves, information is becoming more readily available. This review therefore aims to take a new look at some of those discussions, and Van Leeuwenhoek’s possible experimental methods. Digital photography has made it possible to show exactly what can be seen through his simple microscopes, and how he could have obtained his results by, for example, modifying his microscopes and lighting. Equally, the completion of the series known as the Collected Letters, begun in 1931 with volume 1 published in 1939 and to be completed in 2023, allows researchers to see complete letters in English and modern Dutch. Theories about experimental methods can be tested and the results recorded photographically. Additionally, new, non-destructive techniques such as neutron tomography have improved the evaluation of the authenticity of surviving microscopes.

## Introduction

**Fig. 1:**
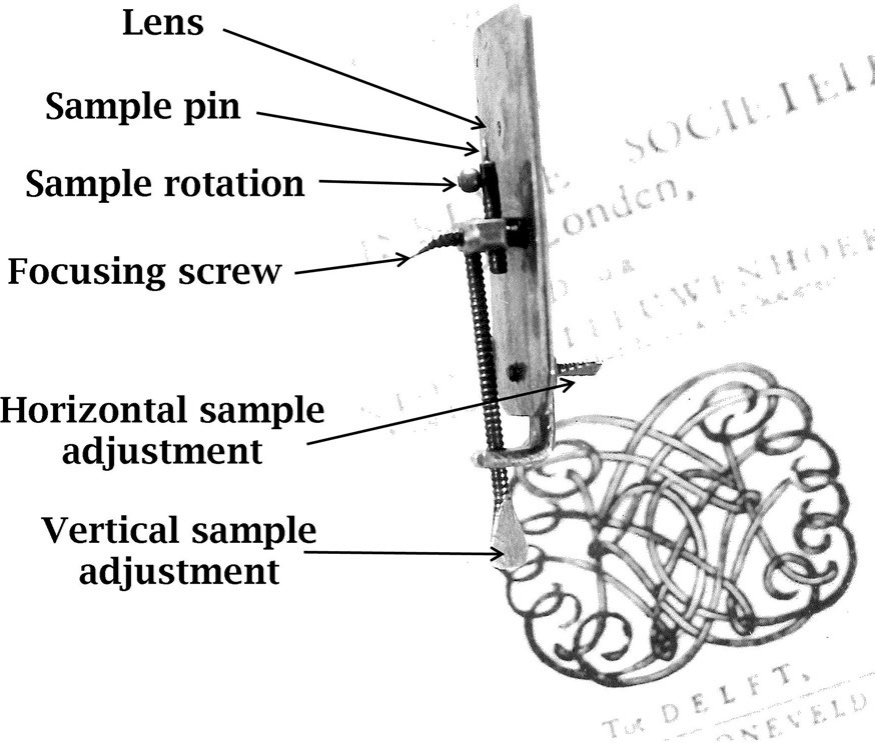
A “standard” Van Leeuwenhoek microscope. (Robertson et al 2016)

On a sunny day in 1674, a young fabric merchant from Delft visited a nearby lake, the Berkelsemeer, and took samples of its unexpectedly cloudy water. As was his habit when he found something interesting, he examined his samples with his simple microscopes (Fig. 1) as soon as he could. His discoveries that day revealed a whole new world, that of microorganisms, and eventually made him famous. His name was Antoni van Leeuwenhoek.

Enthusiastic letters about him from Royal Society contacts, Renier de Graaf and Constantijn Huygens Snr, together with the quality of his work as described in his letters to the Royal Society of London, resulted in his being elected a Fellow of the Royal Society of London on 29 January, 1680 (Birch 1757) along with three others. Van Leeuwenhoek having accepted, the following February:

Dr Gale was called upon for the diploma directed at the meeting of January 29 to be sent to Mr Leewinhoeck, and it was ordered that the Society’s seal should be affixed to it, and that a silver box should be provided for it.

There is no record in the Royal Society Minutes (Birch 1757) of such special treatment for the other three new Fellows. Van Leeuwenhoek was so proud of his Fellowship that he employed Jan Verkolje to paint his portrait with the certificate on the table beside him, and mentioned it frequently in correspondence. On 4 March 1699, he was appointed as a”Correspondent of Burlet “ at the French Academy of Sciences (Academie des Sciences 2023). Claude Burlet (1664–1731) was a botanist and the first doctor to the King of Spain. The earliest Academy membership lists carry a wide range of membership titles, making it difficult to understand their different levels, but in 1699 the Academy had 70 members and 80 correspondents.

There are many published accounts of Van Leeuwenhoek’s life, ranging from Boitet’s chapter (1729) in his book about Delft, written during Van Leeuwenhoek’s lifetime, to last year’s biography by Van Delft (2022). There are more new books in the pipeline as well as reprints of scans of older books, presumably timed to coincide with the 300th anniversary of Van Leeuwenhoek’s death in 2023. Some authors have had better access to original data than others. There are even a couple of fictionalised versions (eg. de Kruif 1926).

This article will review some of the discussion surrounding Van Leeuwenhoek’s life, equipment and work in the light of late twentieth and twenty-first century access to information and fresh experiments using modern equipment. For most of the experimental details the reader is referred to the original publications.

## Who was Van Leeuwenhoek, and how did he reach this key point in his life?

Van Leeuwenhoek was born into a family of basket manufacturers in Delft on 24 October 1632, and his baptism was recorded in the register of the Nieuwe Kerk (New Church) on 4th November, 1632. This was a few days after another of Delft’s famous sons, Johannes Vermeer, was baptised in the same Church on 31 October 1632.

Some authors have assumed that basket making was a lower class activity, even referring to Van Leeuwenhoek as an “artisan” or, as Dobell (1932) put it, “only a basket maker”. It should be remembered that until the beginning of the twentieth century, baskets were an important form of packaging equivalent to modern cardboard or even polystyrene. Delft’s industry at that time included brewing and China production, both of which required packaging. The family was certainly not short of money. The inventory of the Van Leeuwenhoek house made after his daughter’s death reveals a comfortable standard of living and a decent amount of gold and jewelry as well as household accessories (Geesteranus 1745). The most frustrating item is a “box of books” without any other details!

### Education

Van Leeuwenhoek’s education seems to have been quite normal for a middle class boy at that time.

As well as his father being one of the owners of the basket-manufacturing company, many of his mother’s family were involved in different aspects of commerce including fabric manufacture and merchandising. Van Leeuwenhoek’s father died when he was 5, and his mother married Jacob Molyn, the artist and municipal painter, a couple of years later. Van Leeuwenhoek was sent to one of the schools in Warmond, north of Leiden (Boitet 1729). The most likely school was the Latin School run by Cornelis Loveringh. Since Loveringh was a Catholic, the authorities checked to see that the syllabus was suitable for Protestant boys and was presumably not as focused on readying the pupils for the clergy as it would previously have been (Van Seters 1982). During this time, Van Leeuwenhoek should have been taught suitable subjects for a child destined for some form of trade including reading, writing and simple calculation. About 5 years later, Van Leeuwenhoek was apprenticed to a maternal uncle, Cornelis Jacobz. van den Berch, the Sheriff and Baliff in Benthuizen. It was here that the boy may have learned the legal and business skills such as book keeping and accounting (Boitet 1729) which allowed his final instructor, William Davidson in Amsterdam, to report that he had passed the test as a Master of his trade after only 6 weeks (Van Seters 1951).

When he was 16, Van Leeuwenhoek was sent to Amsterdam, probably to stay with another Uncle, Pieter Mauritz. Douchy, husband of his maternal Aunt Catharina. He studied the cloth trade with William Davidson (1615–1689), a wealthy Scottish merchant who was an active supporter of both the Dutch House of Orange and the exiled British Royal Stuarts. His trade empire spread across Europe including salt production in Denmark, iron production in Norway, tobacco from Virginia, and the Scotland-Netherlands trade in wool. He supplied financial and material support to King Charles II before the Restoration in England as well as serving as a spymaster for him in Europe. Sir William (as he became) obviously had faith in Van Leeuwenhoek’s competence since he left him with a Power of Attorney when Davidson had to leave Amsterdam for a period during the first Anglo-Dutch War (Van Seters 1951; Robertson et al 2016).

With this educational background, Van Leeuwenhoek’s frequent statements that he did not speak foreign languages including Latin, French or English might seem strange, but reading a foreign language is often simpler than speaking it, and he certainly had English-language books (eg Van Leeuwenhoek, 1680, Jurin 1722a), and several times mentioned using a Dutch–English dictionary to read the Royal Society’s *Philosophical Transactions* (Van Leeuwenhoek 1676a; 1703).

By the time that Van Leeuwenhoek reached his twenties, he had clearly been given an education suitable for a young man expected to go into trade. He was not yet famous, and any microscopical studies would have been more of a hobby. There was no reason to believe that he would need a different educational direction. Indeed, Van Leeuwenhoek had already started his own haberdashery in the centre of Delft. Why then have some authors (eg. Dobell 1932) considered it strange that he had not attended a University? Where would he have gone? At that time, Universities were few in number, expensive, and mostly attended by men from the upper classes, such as the Huygens brothers. The Universities tended to focus on Faculties of Theology, Law, Medicine and Philosophy (the latter sometimes included topics such as botany and mathematics). Leiden, (inaugurated 1575) was the nearest to Delft. By the time that Leiden University appointed Herman Boerhaave as Professor of Medicine and Botany in 1709 (Underwood 1977), Van Leeuwenhoek was already famous for his discoveries.

## Van Leeuwenhoek, the man

### Marriages

Van Leeuwenhoek married twice. His first wife was Barbara de Mey (1629–1666), daughter of a Flemish serge merchant from Norwich, England. Five children from this marriage are named in the baptismal register of the Old Church, but only one (Maria 1656–1745) survived her first year. Barbara died only 3 weeks after her last son - not named but described as a “baarkind” (probably a stillbirth). Boitet (1729), and a distant relative, Haaxman (1875), both mention a short-lived child born to Van Leeuwenhoek and his second wife, Cornelia Swalmius (1635–1694), a Minister’s daughter. However Van Seters (1968) reported that there is no trace of this child in the records. Barbara’s daughter, Maria, spent her life housekeeping for her father after Cornelia’s death.

### Occupations

As Van Delft (2022) has pointed out, earning a living as a scientist was not an option for most men in the seventeenth century. Unless an individual had a private income or a wealthy sponsor, it was necessary that he also had a paid occupation. After Van Leeuwenhoek returned from Amsterdam to Delft and married Barbara, his business as a haberdasher and fabric merchant must have been the primary call on his attention, although with her family’s background in the textile trade it has been suggested that his wife was also involved in running the business (Robertson et al 2016). In 1660, he also began the first of his appointments for the City Council as the “Chamberlain” (Kamerbewaarder) for the City Aldermen. Dobell provided a translation of the job description (Dobell 1932). In 1669, he was accepted as a surveyor (Boitet 1729; Dobell 1932). In 1694 he wrote about measuring the height of the tower of the New Church (which he could see from his house and the Town Hall) while training as a surveyor. In 1713, he mentioned that his mentor for that exercise was Jacob Spoors (Van Leeuwenhoek 1713), For a time, he was listed as a Curator for the City Council which, among other things, involved sorting out bankrupt estates such as that of Johannes Vermeer. He acted for the Council in at least 8 cases (Robertson et al 2016). From 1666 to 1711, he also served as “Generaal Wijkmeester” (Dobell 1932). Some have described these jobs as “sinecures”, but from the evidence, they were anything but (Anderson 2014; Robertson et al 2016).

### Social contacts & visitors

Van Leeuwenhoek seems to have been a very private man, but that does not mean that he lacked friends. There are very few personal matters discussed in his letters, and even births and deaths among his family or friends are generally only known from the Church records. Even visits by royalty are known about from the accounts of others. Folkes (1723) wrote that Queen Mary visited Van Leeuwenhoek in Delft, used his microscopes with satisfaction and was gifted with two which have not been seen since, although Folkes said that he knew someone who had “*had them in his hands for some time”*. Curiously, in Van Leeuwenhoek’s dedication of one of his books (Van Leeuwenhoek 1693) to the Queen, he apologised for having been out of town when she visited, and therefore having been unable to show her his discoveries. In 1697, Czar Peter the Great visited Delft by boat and invited Van Leeuwenhoek on board to demonstrate his microscopes. The Czar did not want to attract a crowd by visiting Van Leeuwenhoek’s house. The visit has only been described by Van Leeuwenhoek’s friend, Gerard van Loon (1731).

As his fame spread, the number of casual visitors dropping by at Van Leeuwenhoek’s house to see the “little animals” increased and he complained in a letter to Antonio Magliabechi, Librarian to the Grand Duke of Tuscany (Van Leeuwenhoek 1691), that they were taking too much of his time and henceforth would only be admitted if they had introductions from particular people. To the Royal Society, he wrote about visitors sent by Sloane (Van Leeuwenhoek 1710):

all of whom I gladly received, and so will I do all those who have an introduction from Mr Sloane. But if I receive everyone who comes to my house, or tries to come, I should have no freedom at all, but be quite a slave.

To another Fellow from the Royal Society, James Petiver, who had been turned away (Van Leeuwenhoek 1711), he wrote:

I would gladly have received you on divers days; and if you had kept by you the letter from Mr Hans Sloane, you would not have missed a friendly entertainment at my house. And you were sent away especially because you were not known, and because some 8 or 10 days earlier no less than 26 people came to see me within four days, all of them with introductions (except a Duke and a Count, with their Tutor): which made me so tired, that I broke out in a sweat all over.

The added pressure from visitors expecting to be given microscopes during their visits could not have helped his stress levels.

Douglas Anderson (Anderson 2023a, b) has identified visitors who are mentioned in Van Leeuwenhoek’s correspondence with the Royal Society. Many were foreign folk on “Grand European Tours”. Others had genuine scientific interests, such as Richard Bradley, the first Professor of Botany in Cambridge. Bradley went on to formulate an early form of Germ Theory based on his botanic studies, long before “germs” had been fully recognised:

…we may observe, that Mankind, Quadrupeds and Plants seem to be infected in the same manner, by unwholesome insects*; only allowing this difference, that the same Insect which is poisonous to Man, is not so to other Animals and Plants. All Pestilential distempers, whether in Animals or Plants, are occasion’d by poisonous insects convey’d from Place to Place by the Air

* He used ‘*insects’* as a synonym for ‘*animalcules*’ (Bradley 1721; Robertson 2022).

Another notable was Zacharias Conrad von Uffenbach (1754), a German who spent much of his life touring European scientific collections and libraries with his brother, describing the collections and making catalogues. His accounts of his travels were published after his death, and included what is believed to be the first published drawing of Van Leeuwenhoek’s microscope. Other visitors, including some who might be described today as “celebrities”, are discussed elsewhere (Robertson et al 2016; Van Delft 2022).

An alternative reason for his suspicion of visitors later in life might have been concern about plagiarism. For example, when Hartsoeker tried to visit his house with the Mayor of Delft (having asked the Mayor not to mention his name) Van Leeuwenhoek realised who he was and refused to allow him to see anything (Robertson et al 2016). The life-long war of words between the two men appears to originate from their youth when Hartsoeker visited Van Leeuwenhoek in the company of his father. Van Leeuwenhoek allowed them to see his spermatozoa samples, and Hartsoeker then showed similar preparations to Christian Huygens and others in Paris, claiming the discovery.

Among those that Van Leeuwenhoek thanked for hospitality at their homes was the diplomat and negotiator of the Treaty of Utrecht, Baron Frederik Adriaan van Reede van Renswoude and his wife, Maria Duyst van Voorhout. Maria was the daughter of one of the Delft Mayors, a neighbour and long term friend of Van Leeuwenhoek’s. She bought 4 of Van Leeuwenhoek’s microscopes at the auction (Rees 1747), and he dedicated one of his books to the Baron (Van Leeuwenhoek 1696).

## Microscopy

Van Leeuwenhoek did not invent the microscope. The earliest known magnifying lenses date from the Assyrian empire of around 700BC and then Egypt, Greece and Babylon. Some were made from polished crystals, others of flasks of water or precious stones and they had a range of uses from fire starting to enlarging delicate carving (Robertson et al 2016). In Europe, interest in magnification increased during the sixteenth and seventeenth centuries and there are surviving publications involving low level magnification of insects (eg. Muffet 1634). Seventeenth century microscopes fell into two groups -“simple” microscopes which only had one lens, and “compound” microscopes with two or more. They each had advantages and disadvantages.

**Fig. 2:**
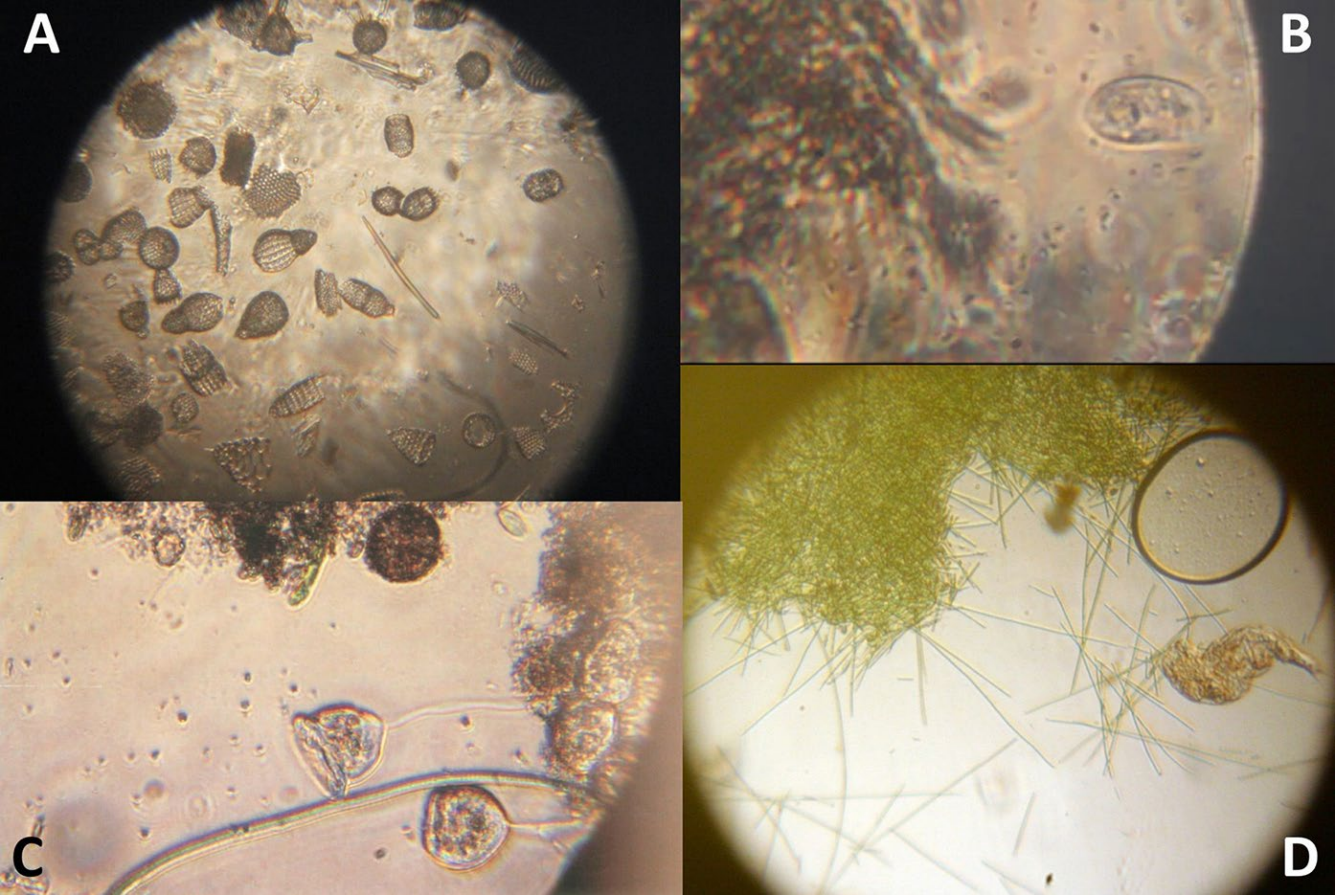
Photographs taken with facsimile  Van Leeuwenhoek microscopes. **A** Fossil diatom mixture, using a microscope with 65 × magnification, a Canon EOS 60D camera body and a Tamron zoom 1:1 macro lens. **B** Bacteria and protozoa, using a microscope with 302× magnification, a Canon EOS 60D camera body and a Tamron zoom 1:1 macro lens. **C** *Vorticella* sp. from a stream in the Delft Botanic Garden. Microscope with 116× magnification, a Canon EOS 550D camera body and a Tamron zoom 1:1 macro lens. **D** Cyanobacteria and a rotifer in a sample of water from the Delftsehout, a shallow lake near Delft. Microscope with 116× magnification, a Canon EOS 550D camera body and a Tamron zoom 1:1 macro lens. (Robertson 2015a)

Robert Hooke used a compound microscope to prepare the illustrations for his famous book, *Micrographia* Hooke (1665), although he also mentioned simple microscopes and how to make them. Van Leeuwenhoek only used the single lens version (Fig. 1) for all of his work. Figure 2 shows images that were obtained with modern facsimiles of his microscopes and a digital camera. When he became aware of Van Leeuwenhoek’s work, Hooke made a comparison of single and compound microscopes and concluded that compound microscopes generally easier to manipulate, but adding extra lenses caused image distortion. Simple microscopes were difficult to operate, especially as their strength increased, but with only a single lens, the quality of the image was better (Hooke 1679). Many of the microscopes made for other users were beautiful as well as functional, with attachments—a mirror, a holder for multiple samples, or even ornamental engraving. George Adams produced the most elaborate designs of both single and compound microscopes (Adams 1746), leading John Mayall to comment that Adams’ simple microscope was very inconvenient as its light weight required the user to continually hold it upright with one hand while focussing with the other (Mayall 1886). Microscopes were often professionally made to the specifications of a particular user, and varied with the requirements of that individual. As time went on, microscopes were included in commercial optical catalogues such as that issued by the Van Musschenbroek company of Leiden (De Clerq 1997).

Van Leeuwenhoek microscopes are unusual because he made them exclusively for his own use. Many people describe them as primitive or crude, but he only required a way to hold a sample in front of a lens while adjusting the focus and view, and his simple microscopes fulfilled his need.

### A closer look at Van Leeuwenhoek’s microscopes

The”standard” Van Leeuwenhoek microscope is characterised by a small lens mounted in holes between a pair of flat metal plates, with an attached pin to mount a sample (Fig. 1). With many samples (drops of liquid, tissues, insect parts, etc.) light is passed through the sample and the lens to the observer’s eye (bright field microscopy). However, lighting the sample from the other side (“top lighting") is difficult as the metal lens plate shadows the sample. Suggestions that he made some microscopes from polished silver to reflect light on to the sample or used a “Lieberkuhn”- type reflector for the same purpose do not work (Robertson 2017). Larger lenses (generally with lower magnifications) mounted in a simple metal ring do not have this problem.

**Fig. 3:**
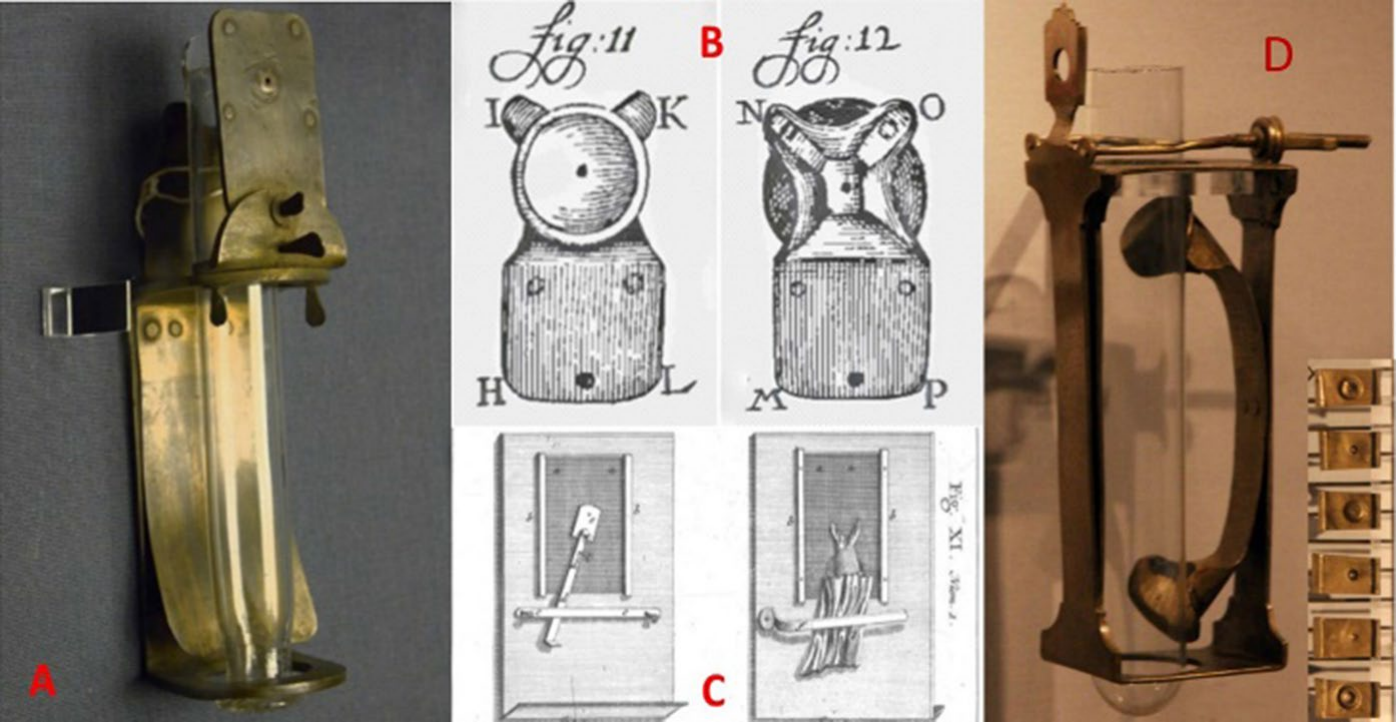
Van Leeuwenhoek’s various modifications of his “*aalkijkers*”. **A**: The “standard” version with a glass tube for water to keep the eel or fish alive. **B**: The minimised eyepiece with eye-protecting cup holding the lens, which can replace the rectangular microscope plate. **C**: The “flat” version where the eel is wrapped in wet fabric and held against a glass plate with the microscope on the other side of the glass. **D**: Modification of A with (bottom right) a changeable lens plate (modified from Robertson 2017)

Van Leeuwenhoek was not inflexible where his tools were concerned, and twice he wrote about modifying his microscope so that he could observe the passage of red blood cells through the capillaries in the tails of living fish or eels (Van Leeuwenhoek 1695, 1710), and also show it to visitors. In place of the sample pin on his microscope, he attached a glass tube in which his fish or eel could be held head down in water (Fig. 3A). Figure 3B shows a lens mounted on a much-reduced microscope plate to try and light the sample from the observer’s side. The cups are to protect the eye. To make observation easier for visitors, he made a second version where the fish was wrapped in wet fabric and then mounted against a piece of flat glass. The microscope lens plate was then mounted on the other side of the glass Fig. 3C). Figure 3D shows an arrangement similar to 3A but with a holder that allowed lenses to be changed, giving different magnifications. English language names for these microscopes vary, so for convenience the Dutch term “aalkijker” (eel watcher) will be used here.

**Fig. 4:**
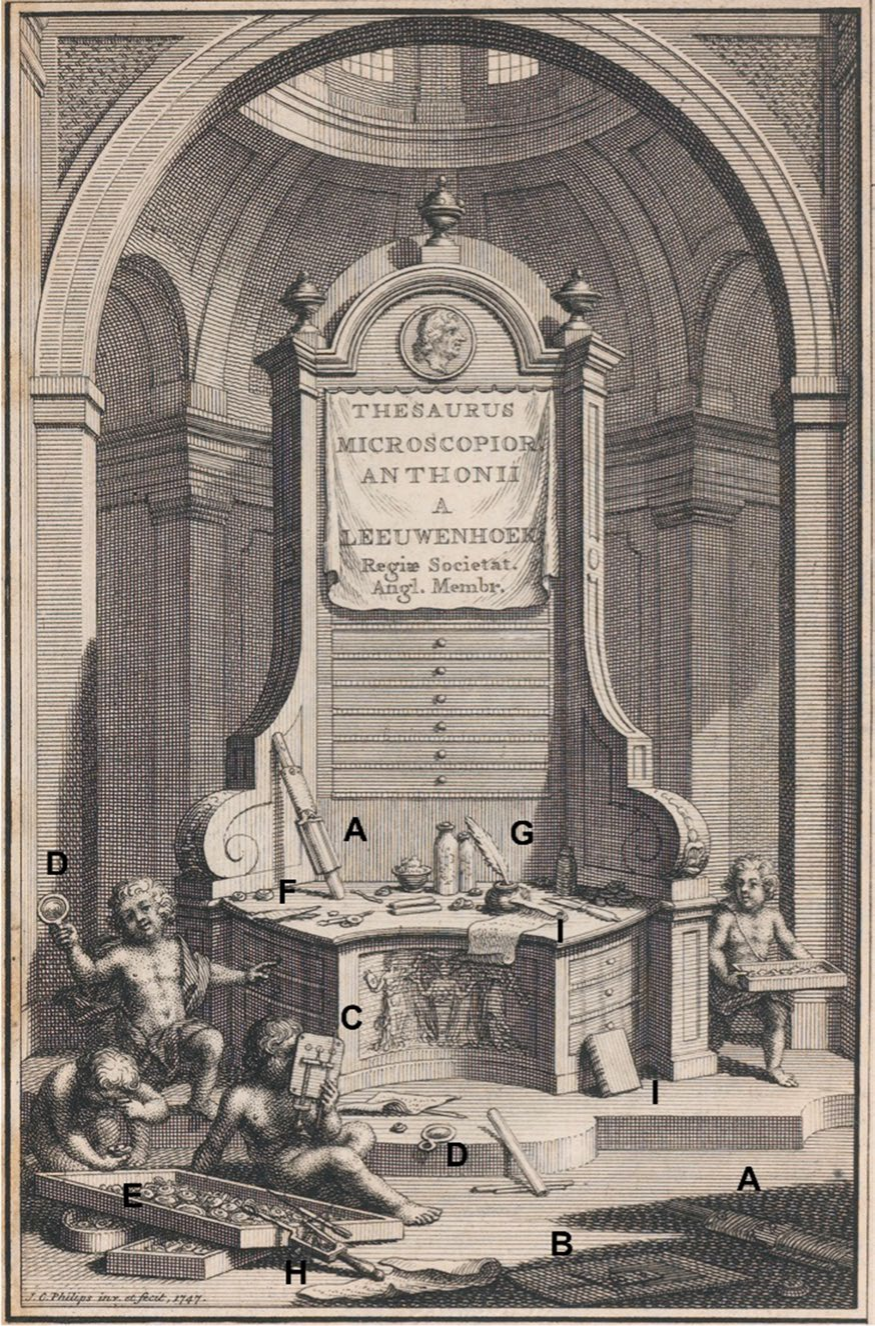
Frontispiece from the catalogue for the sale of the microscopes (Rees 1747). **A** original aalkijker; **B** newest form of aalkijker; **C** three-lensed microscope; **D** magnifying glass; **E** loose lenses; **F** tweezers; **G** quill pen and ink; **H** possibly microscope with weaker lens; **I** bound book. (Robertson 2015c)

Van Leeuwenhoek modified his “standard” microscope again, this time by adding extra lenses to microscope plates side by side. Most samples are fragile and mounting them in glue on a sample pin is time consuming and can be destructive. It was easier to move the sample from one lens to another on the same lens plate rather than transfer it to another instrument. Microscopes with three lenses side by side can be seen on the frontispiece of the microscope auction catalogue (Fig. 4c), as well as in the well-known portrait by Verkolije (1686 Fig. 5a). Others with two lenses are mentioned in the auction catalogue. The catalogue frontispiece shows examples of the various tools mentioned in the catalogue.

**Fig. 5:**
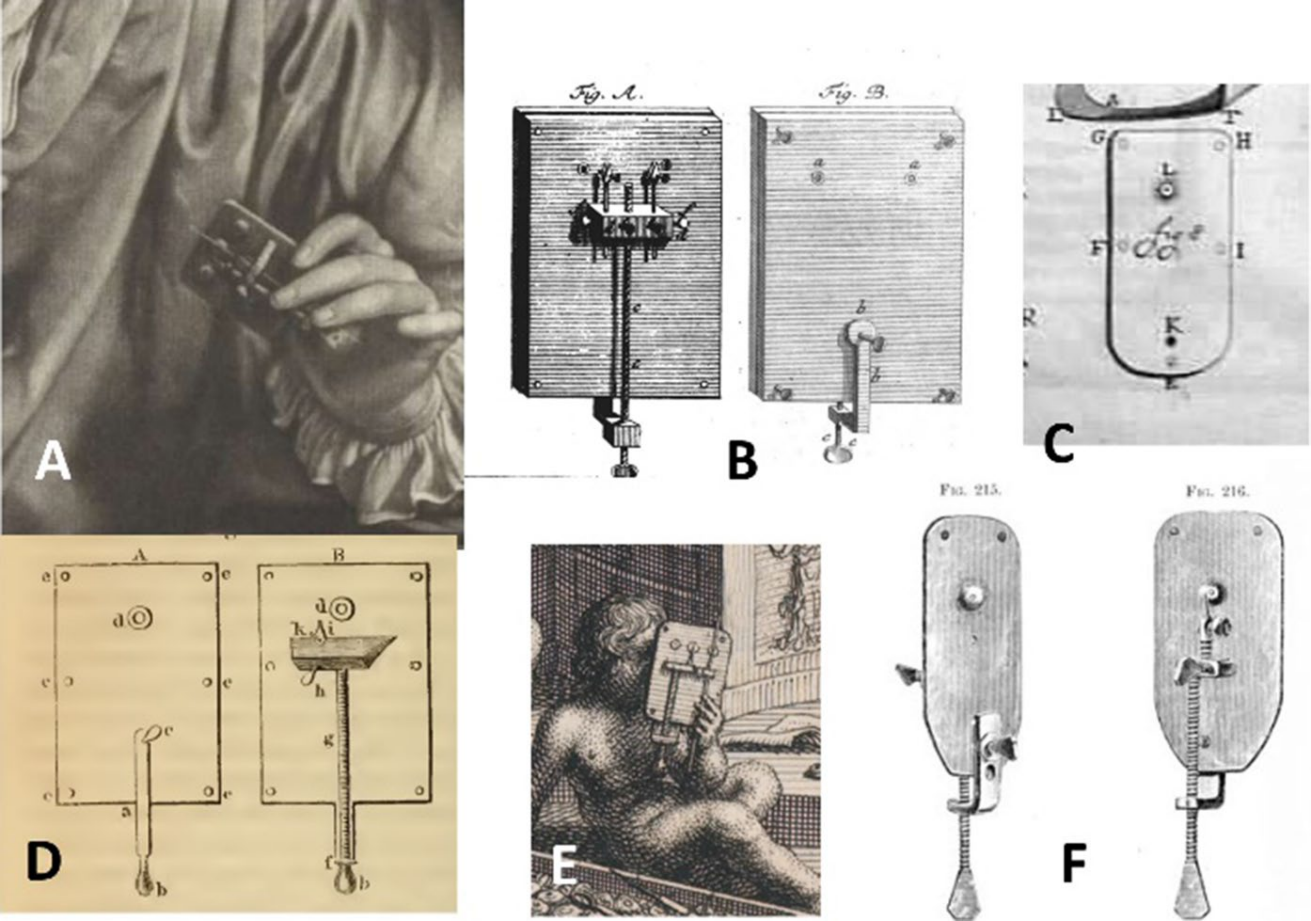
Historical drawings showing modifications of Van Leeuwenhoek’s “standard” microscope. These are all details of microscope plates from historical drawings **A**: 3 lenses and a centrally-placed capillary, Verkolij (1686); **B**: 2 lenses and right angle corners on the lens plate, Von Uffenbach (1754); **C**: Small drawing of “standard” lens plate with single lens, Van Leeuwenhoek (1685); **D**: Single lens and right and corners on lens plate, Baker (1742); 3 lenses **E**: 3 lenses and a capillary to the right, Philips (1747); **F**: Exact drawing of the Utrecht microscope, Mayall (1886)

Van Leeuwenhoek’s lens making methods have, down the centuries, also been a matter of discussion.

Delft seems to have been a centre for optics since before Van Leeuwenhoek’s birth (Zuidervaart & Rijks 2014) and several lens making techniques would have been available to him. He generally made them from glass, but a few were made from minerals such as quartz (Rees 1747).

Obviously the latter were ground, but was this also true of all of those made from glass, particularly the stronger ones? His apparatus for grinding lenses is mentioned in one of his letters (Van Leeuwenhoek 1676b) and also in the inventory of the Van Leeuwenhoek house made after his daughter’s death (Geesteranus 1745). In 1694 he wrote that that his glass blowing skills were limited, having learned by watching a demonstration by a glass blower at a fair in Delft. Johannes Hudde had developed a popular method of producing numbers of flameworked, solid balls (Bolt et al 2018), but when the Von Uffenbachs asked Van Leeuwenhoek whether he used them, he pointed out that the two lens plates on his microscope were so close together that they required a biconvex lens to fit between them (Dobell 1932). Van Leeuwenhoek later commented that he had stopped making very tiny lenses and he considered that for research.

Those which had been ground to a larger diameter are more suitable (Van Leeuwenhoek 1699).

This preference is reflected by the dominance of microscopes with roughly 100× magnification in the microscope auction catalogue and Folkes’ description of the Royal Society bequest (Folkes 1723; Rees 1747). However, his lenses provide further evidence of Van Leeuwenhoek’s willingness to adapt his methods of making his tools as required. Using neutron tomography and authentic microscopes, Cocquyt et al (2021) have shown that while a weaker lens was ground and polished, the lens in Utrecht University’s very strong microscope is flameworked in a manner similar to that described by Robert Hooke (1665).

Lighting problems related to shadowing were mentioned above, but there was also the question of whether or not Van Leeuwenhoek was able to light samples for “dark field microscopy” with such a simple microscope, as suggested by his 1675 description of red blood cells (Dobell 1932):

. . . but I can demonstrate to myself the globules [= corpuscles] in the blood as sharp and clean as one can distinguish with one's eyes, without any help of glasses, sand grains that one might bestrew upon a piece of black taffety silk.

**Fig. 6:**
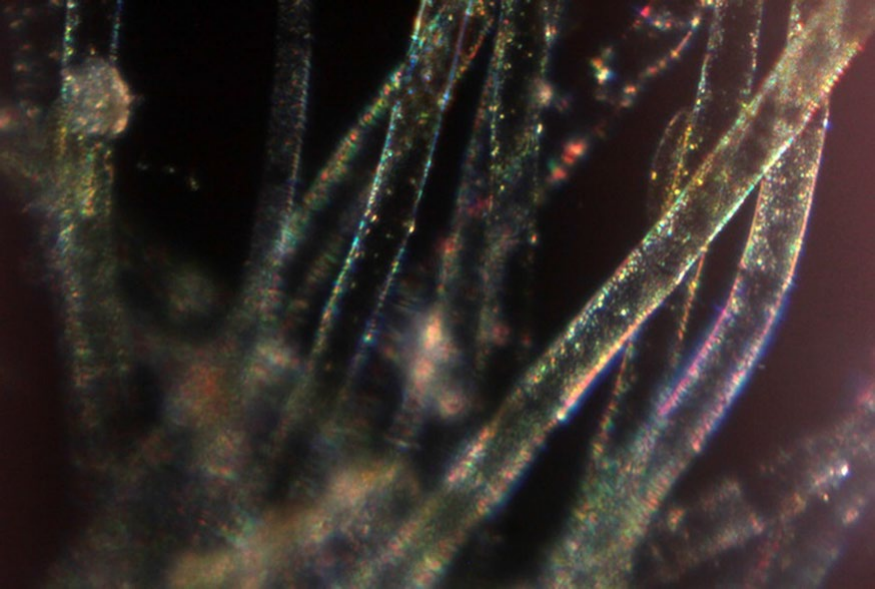
The first dark field image obtained during a lighting control experiment using a facsimile microscope and an unknown algal sample. (Robertson 2015a)

**Fig. 7:**
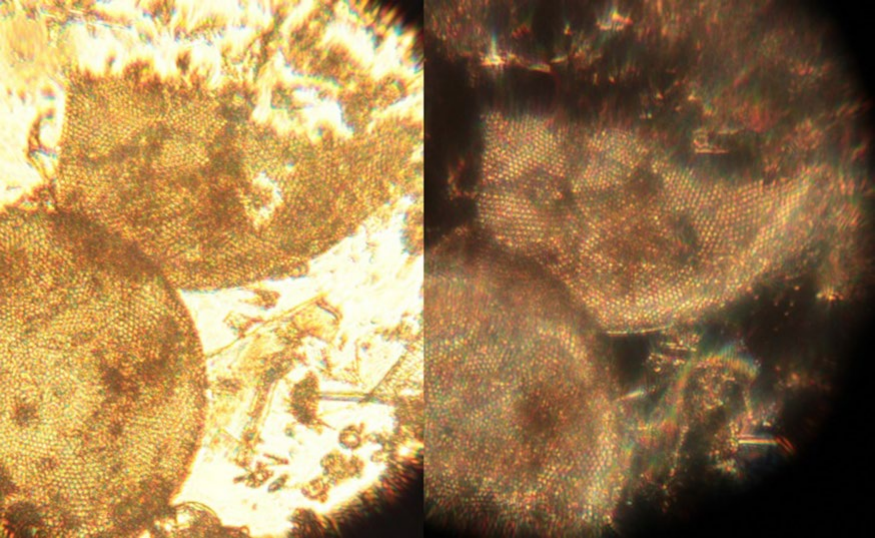
Comparison of bright (left) and dark (right) field lighting of a fossil diatom preparation using a microscope with magnification 302× , a Canon EOS 550D camera body and a Tamron zoom 1:1 macro lens and the lamp used for Fig. 2C. (Robertson 2017)

Serendipity revealed that dark field microscopy is indeed possible. When a facsimile microscope being used to demonstrate that a magnifying glass in the light path improves lighting control was accidentally turned in place through about 45˚, the change in the light path combined with a dark background revealed that it was comparatively simple to do (Fig. 6), but was not necessarily better (Fig. 7) (Robertson 2017).

Sample mounting was obviously determined by the structure of the sample. Many samples that could support themselves (wood, dry tissue, insect parts, etc.) were usually attached to the sample pin with a small drop of glue. Some fragile samples such as bee and dragonfly corneas needed to be attached to a supporting surface such as a mica flake (muscovy glass) or glass (Robertson 2019). However, drops of water or other liquids required some sort of containment, and Van Leeuwenhoek often mentioned using small glass tubes (now known as “capilleries”), something that Hooke seems to have found inconvenient (Hooke 1679). The microscope shown in the Verkolje drawing (Fig. 5A) mentioned above includes a holder for a thin tube (or capillary) (Robertson 2017). Capillaries are not always convenient (eg with particulate samples) and Van Leeuwenhoek also mentioned using drops of liquid on mica flakes or thin glass (Van Leeuwenhoek 1679). In modern experiments, it has been convenient to replace the mica with glass cover slips which gave similar results.

**Fig. 8:**
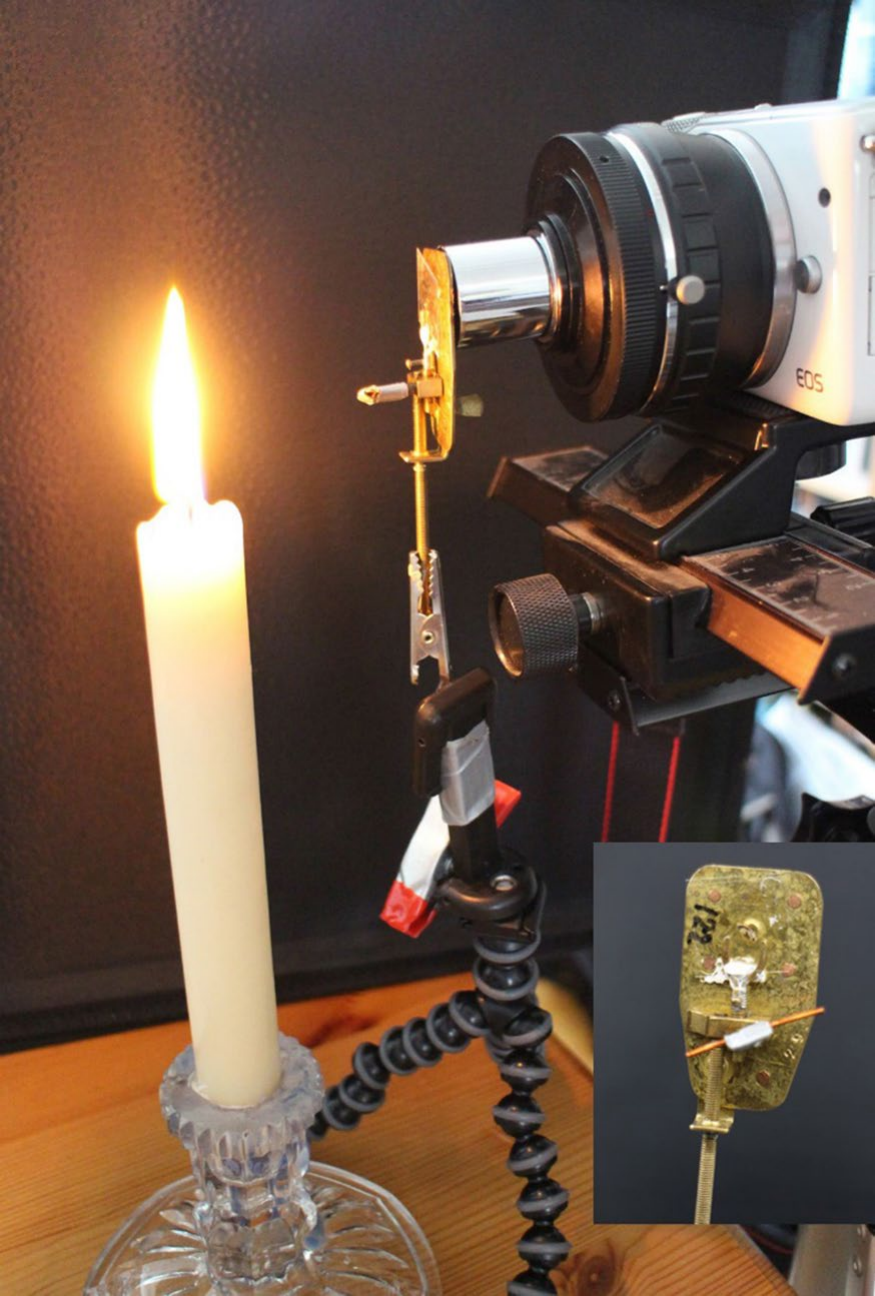
Setup used for photography and filming with the facsimile microscopes and an eyepiece adaptor in place of the macro lens used in other experiments. From left to right: candle as light source, facsimile van Leeuwenhoek microscope in clamp, Canon EOS M10 camera body fitted with eyepiece adapter. Inset: the back of the microscope with copper wire attached to aid fine focus. Robertson (2019)

The focus screw is reasonably convenient to use but it does require firm support for the microscope and can be unstable when held in a tripod for use with a camera. It has been easier to add a small piece of copper wire across the focus screw head, increasing the turn of the screw and thus the focus sensitivity (Fig. 8).

Van Leeuwenhoek seems to have been a pragmatist who made and used whatever was necessary to see whatever he was working on without worrying about ornamentation. Of course, each of the different designs for single microscopes in use by others at the time had its own advantages and limitations, as experiments with an original Van Muschenbroek microscope revealed (Quint and Robertson 2019). Sadly, facsimilies of these other simple microscopes are not as readily available for modern experiments as the Van Leeuwenhoek ones have been.

Some researchers have followed his pragmatic approach when teaching or examining his results. They have concentrated on the use of single magnifying lenses by devising different ways of holding the lenses and samples without the limitations of Van Leeuwenhoek’s microscope structure.

As Cocquyt (2022) has shown, evaluation of Van Leeuwenhoek’s microscopy and the development of his tools must be done against the background of the work of his predecessors and contempories. He was obviously influenced by the Royal Society’s requests (eg for attention to be paid to sperm or the contents of different blisters) and commented on what he found in the Philosophical Transactions, with the help of an English-Dutch dictionary (see above).

### Samples

When reading through Van Leeuwenhoek’s published work, one cannot help but be impressed by the range of sample types that he managed to examine under magnification. Speciman size was obviously a concern, but so was opacity, whether specimans were alive or dead and whether a sample could be lit appropriately. (Robertson 2017). Of course, the reverse is also true. Introducing a camera to the light path of an experiment immediately introduces depth of field problems that increase with the increasing magnification power of the lens (Fig. 2B).

Metrics: Defining the relative sizes of things that he saw in his samples was a recurring problem that he did his best to standardize by comparison with every day items such as a hair from his beard or fine sand. James Jurin of the Royal Society (Jurin 1722b) was enthusiastic about his techniques, but also suggested an alternative method as a way of standardisation, using fine silver wire. He asked Van Leeuwenhoek to try this method, and even sent some of his own wire to Delft. Van Leeuwenhoek’s results agreed well with Jurin’s. Ian Davis (2020, 2022) has reviewed many of his calculations, and shown that Van Leeuwenhoek’s numbers are still relevant today.

Douglas Anderson has summarised many of Van Leeuwenhoek’s samples from his letters (Anderson 2023a, b). They include plant structures, animal tissues ranging from insects to whales, minerals and salts. Captains and crew from the Dutch East India Company brought him samples they had collected and sometimes Dutch colonists sent material. Of course, he collected many himself, and others were supplied by local people. One of his most famous lines of research, spermatozoa, began in 1677 when a Leiden Student, Johan Ham, brought a sample containing animalcules from one of his patients who had “*lain with an unclean woman*” (Van Leeuwenhoek 1677). With encouragement from the Royal Society, Van Leeuwenhoek investigated further and was able to show that all males (including himself), no matter the species, had them. They were obviously nothing to do with the patient’s disease. He took other samples from his own body including material from between his teeth (which produced one of the first drawings of bacteria) and toes, and sometimes from his daughter and maids as well as from tramps off the street. He and his wife incubated samples in their pockets, and he complained when they began to smell (Leeuwenhoek 1687)**.** It appears that he always had sample bottles with him, hence his sampling of the Berkelsemeer.

He did not hesitate when an interesting sample presented itself. For example, when a dragonfly happened to land on his sleeve, he caught it and, having removed its head:

I cut off the Eyes of this little Animal (a dragonfly) and laid them on a clean piece of paper, and then with a small brush I cleared away with clean rainwater the numerous vessels from the Cornea of the Eye on the inside in such a way that hardly anything but the Cornea of the Eye was left…

**Fig. 9:**
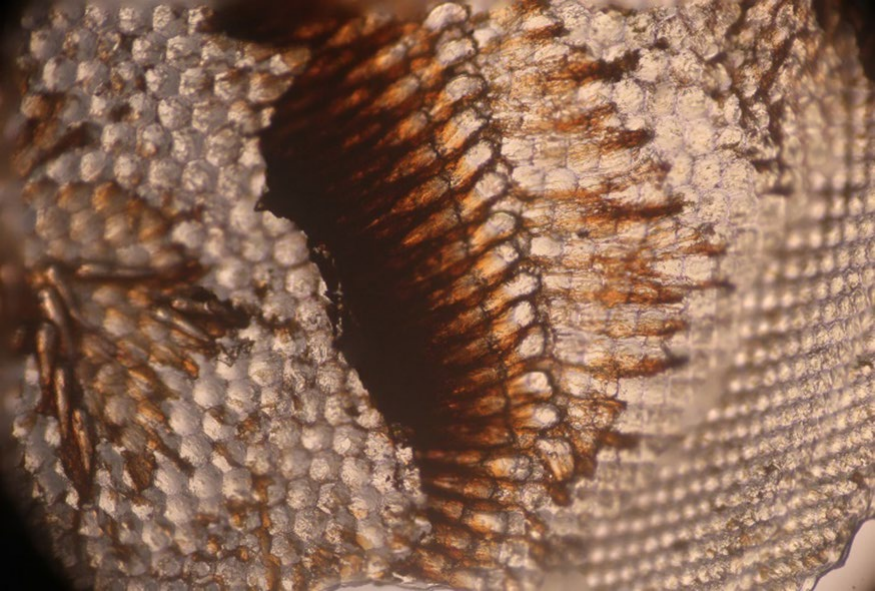
Dragonfly cornea photographed using dark field lighting to show the ommatidium patterns as well as the lenses

I placed it (the cornea) before the microscope, and I often contemplated it with great admiration. (Fig 9)

Van Leeuwenhoek’s descriptions of finding samples have supported the principle that repeating historic experiments (“living history”) is useful provided that care is taken with experimental detail. Speculation and theory are not sufficient. For example, there is at least one modern illustration of Van Leeuwenhoek holding his microscope to his eye in a “landscape” orientation, despite the fact that it is very difficult to stop preparations falling off. It is also uncomfortable. Again, working from Van Leeuwenhoek’s text, Dobell (1932) identified the algae that Van Leeuwenhoek called “green streaks” as *Spyrogyra*, something that was accepted by many others. However, a combination of ecophysiology and photography has now allowed Van Egmond (2016) to make a more likely identification as the cyanobacterium *Dolichospermum*).

**Fig. 10:**
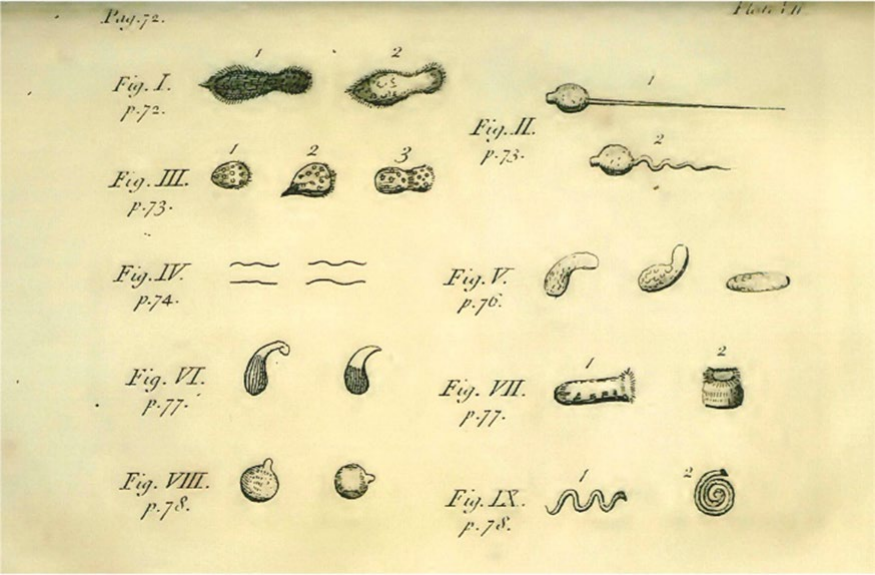
Copy of the drawing that accompanied Van Leeuwenhoek’s letter about his pepper water experiment (Van Leeuwenhoek 1676b). The original drawing has not survived, but Henry Baker published his version as an exact copy (Baker 1742). “Figure IV” among the various protozoa on the image is regarded as the first published drawing of bacteria. (Robertson et al 2016)

Sometimes, researchers also still claim that he could not have seen bacteria with his usual microscopes, despite the illustrations (eg Fig. 10) that accompanied his letters and that they have now been filmed (Robertson 2014). As mentioned above, photography through the stronger Van Leeuwenhoek facsimilies has been limited by the available depth of field when combined with a camera (about “half a bacterium”). It is sometimes easier to film a preparation and then cut out a suitable frame if one is needed. The view is better when the microscope is used without a camera or with a weaker lens (Robertson 2014).

## 20th/21st century experimentation

As mentioned above, “living history”, where researchers or TV presenters use historical equipment such as agricultural implements and kitchen implements or adopt domestic lifestyles from the past rather than theorizing, can provide a great deal of useful information and eliminate (or even confirm) theories. It has become now popular in archaeology and agricultural history, among others subjects. “Historical microbiology” is a limited form of this where historically important experiments are repeated with original equipment or accurate copies to test how such equipment could have been used in the days before method descriptions were routinely included in publications (Robertson 2015a) or removed by editors (eg. Robertson 2019). One of the essentials for such work is obtaining useable original instruments or accurate copies for the experiments. It seems reasonable to expect that repeating selected experiments from Van Leeuwenhoek’s work while using photography to compare results with his drawings should also shed useful light on his methods, results and what he could actually see (e.g. Fig. 2).

Repeating one or two of Van Leeuwenhoek’s experiments has also proved popular in teaching. For example, in Delft, first year students are provided with the letter describing the finding of “animalcules” when peppercorns are soaked in water from different sources, and they are challenged to work out (within the limits of a modern laboratory) how he did it. Not only does this experiment comply with biosafety regulations (enrichment of bacteria on a mix of pepper and water is unlikely to enrich for organisms higher than level 1), but the students enjoy the research (Robertson 2015a).

## Authentic microscopes and facsimiles

Of course, with microscopes of such simplicity, accurate facsimiles can be made, the lens quality being of the greatest importance for use (see for example Loncke 2006a; b). However, accurate copies of Van Leeuwenhoek’s microscopes do not seem to have been available until relatively recently. There was a 1702 advertisement for John Marshall, a well- reputed maker and seller of optical instruments at the “The Archimedes and Spectacles” (Clifton 1996), which included microscopes “according to Mr Leewenhoek” (Salmon 1702), but the drawings by Baker (1742) and Von Uffenback (1754) seem to have been the first published (Fig. 5b, c) The shapes of their microscope plates were very distinctive and would have been obvious if someone had copied them. Moreover, had copies been available, they would surely have been mentioned in the minutes of the Royal Society at that time, considering the Members’ interest in how the microscopes were made, and Marshall’s regular contact with the Society? Mayall (1886) made a few exact copies of the authentic microscope owned by Utrecht University, and published an accurate scale drawing of it (Fig. 5e). These copies are now museum pieces in their own rights and not available for extensive experiments. The Rijksmuseum Boerhaave in Leiden began selling copies of one of their authentic microscopes in the late twentieth century, and now sell an improved version. Others have also made good copies, including Hans Loncke, who made the accurate facsimiles (with ground lenses) for the experiments described in this article (Loncke, 2006a; b).

The Dutch film maker, Jan Cornelis Mol, helped by W.H. van Seters, made a film about Antoni van Leeuwenhoek in 1924, including the first brief sequence showing live microorganisms filmed through an authentic Van Leeuwenhoek microscope. However, the equipment was cumbersome, and original microscopes are both fragile and extremely valuable. Photography and filming with Van Leeuwenhoek microscopes (and facsimiles) became much simpler in the late twentieth century with the appearance of digital cameras (Robertson 2014).

Some researchers find it convenient, especially when teaching, to concentrate on the possibilities of single lenses, and not worry about the complications of pin-mounted samples and small focusing screws so they have used simple lens holders instead (eg. Flores and Marzullo 2021).

In 1981, Ford published an account of his work on authentic Van Leeuwenhoek cork samples from the Royal Society as well as modern bacterial preparations, comparing results obtained with a Wilson screw barrel microscope, modern microscopes and the strongest surviving Van Leeuwenhoek microscope. As part of a sample preparation study, he also used modern stains. Van Leeuwenhoek sometimes used saffron in cognac as a stain for some cells and tissues (Van Leeuwenhoek 1714), but thus far, modern saffron samples have not given a very strong reaction with bacteria or yeast (Robertson et al 2016).

Inspired by a brief visit to Delft by a BBC team to film a sequence about Van Leeuwenhoek’s work with sperm for “The Cell” (BBC 2011), Lesley Robertson began a long-term series of experiments to repeat some of Van Leeuwenhoek’s work using facsimile microscopes made by Hans Loncke and digital cameras. Many of the results presented here came from this series. A short film showing some of the results won the FEMS International Image Contest in 2014 (FEMS 2014). Figure 8 shows the setup used for recent experiments, when a telescope eyepiece adaptor replaced the macro lens used in earlier experiments.

Recently (Press Release 2019), the Royal Society has collaborated with the Rijksmuseum Boerhaave to examine other samples left by Van Leeuwenhoek, using an authentic Van Leeuwenhoek microscope, making both photographs and film clips. The specimans were cork sections and elder pith, optic nerves from cows, cotton seeds and algae mats. One of the photos of the cow optic nerve made worldwide news headlines. In 2021, the collaboration was extended with a 6-year study involving a number of European institutions, called “Visualizing the Unknown in 17th-century Science and Society” (Visualising the Unknown 2023). 

### Authentication of microscopes

A modern problem associated with Van Leeuwenhoek’s microscopes is their authentication. Van Leeuwenhoek left about 350 completed microscopes as well as more lenses set in “aalkijkers” and brass holders. Almost all have vanished. As mentioned above, the earliest known published drawings of them were those published by Baker and von Uffenbach and show a distinctive, almost square instrument with rectangular corners and multiple lenses side by side. Because of their simplicity, any copies made from these drawings would be immediately obvious.

After John Mayall published his accurate microscope drawings of the Utrecht microscope (Mayall 1886), it became simpler to make copies. Well-known copies were reviewed by Robertson (2015a). They include some based on the Utrecht microscope by Filibri and others based on one of the Haaxman microscopes at Museum Boerhaave in Leiden, all of which are marked as copies. Previous identification often depended on physical examination and the idea that since the microscopes were handmade, they should not resemble each other too closely (van Zuylen 1981; Robertson 2015b). Modern makers generally mark their products.

Cocquyt (2015) summarised available evaluation techniques when an apparent Van Leeuwenhoek microscope was found among an English collection of furniture for a doll’s house, and was able to authenticate it by comparison with a range of physical references including silver marks. The chemical content of modern silver is different to that of silver items from Van Leeuwenhoek’s time because of their silver extraction processes (Cocquyt 2015). Subsequently, non-invasive methods such as neutron tomography for analysing the metal and glass have appeared (Wassink 2018), and authentication is becoming less subjective (Bolt et al 2018, Cocquyt 2022; Cocquyt et al 2021). For example, the facsimiles used for the photography and filming shown here deliberately resemble various authenticated microscopes as closely as possible, even including the method used to grind the lenses (Loncke 2006a; b). However, analysis of the materials they are made from will immediately reveal that they are modern copies. Not only do their physical measurements closely match authenticated microscopes, but the chemical constitution of the metal structure is modern, as is the glass of their lenses. They are also numbered, of course.

## The final years

Van Leeuwenhoek was not always correct in his conclusions about his results, and often fiercely defended his ideas. For example, he was quite convinced that rather than spontaneous generation happening, sperm from males were the sole causative agent of embryos, with females serving as incubators (Van Leeuwenhoek 1677). He referred to “ovaries” as “testicles” whose function was to supply nutrients to the child developing from the sperm.

…If your Harvey and our de Graaf had seen the hundredth part they would have stated as I did that it is exclusively the male semen that forms the foetus and that all that the woman may contribute only serves to receive the semen and feed it...

Again, despite other researchers making the connection within a couple of years of his discovery of microorganisms being published (Robertson 2022), he did not believe that his animalcules caused disease. After a query from Hans Sloane and James Jurin at the Royal Society because of their growing interest in inoculation, he replied in July 1722 (Rusnock 1996) with his reasons for not believing that animalcules cause small-pox or other diseases. He seems to have considered all blisters (or pustules) on the skin as having the same cause, whether they were associated with sunburn, cold or infections. He thought that they were caused by thickening of the blood and blockage of the small blood vessels, which could be relieved by fever. However, he was willing to compare the contents of Itch blisters (scabies) with those of measles and smallpox as soon as the local orphanage could provide children with suitable infections (Rusnock 1996). Of course, measles and smallpox are both caused by viruses, which he could never have seen with his microscopes. That required a couple of centuries and an electron microscope. This was to be the last letter sent to the Royal Society during his lifetime, and word of his death arrived in a letter dated 30 August 1723 to James Jurin from Peter Gribius, Minister of the New Church in Delft (Dobell 1932; Rusnock 1996).

Van Leeuwenhoek had continued his studies until his last days, when he dictated two letters and then gave them to a friend, Jan Hoogvliet, to translate and send to the Royal Society. The December 31st 1723 issue of the Philosophical Transactions included 4 letters relating to Van Leeuwenhoek’s death. The first was from Hoogvliet (Dobell. 1932):

Our venerable old man Leeuwenhoek, already dying, and nevertheless mindful of his art, ordered me to be called to him, and lifting up his eyes, already burdened by death, he asked me in half-broken words, if I would like to translate these two letters from the vernacular into a Latin sermon, and to send them to you.

Do not indent, not part of the quotation immediately above. Van Leeuwenhoek’s last 2 letters followed. The first described spheres observed in blood and wine (Van Leeuwenhoek 1723a) , and the other concerned the generation of animals and the palipitation of the diaphragm (Van Leeuwnhoek 1723b).

The last letter was a detailed description by Folkes (1723) of the microscopes bequeathed to the Royal Society by Van Leeuwenhoek.

Van Leeuwenhoek’s epitaph on his memorial in the Old Church in Delft includes a flowery verse (in the style of the time). Boitet (1729) published 2 long poems (by Hendrik Schim and H.K. Poot) and other verses in praise of him. However, 300 years later, the simplicity of his own words somehow seems a more appropriate memorial:

From a quotation attributed to Van Leeuwenhoek (de Kruif, 1926):

People who look for the first time through a microscope say, 'Now I see this, and then I see that,' and even a skilled observer can be fooled. On these observations I have spent more time than many will believe, but I have done them with joy, and I* have *taken no notice of those who have said, 'Why take so much trouble,' and, 'What good is it?'

And from Van Leeuwenhoek’s 1716 letter to Antoni Cinck:

I did not work for more than 40 years in order to gain praise, but because of the curiosity that is strong in me. When I found something worth noting, I considered it my duty to write my observations down so that the educated world can also know about them.

RIP Antoni van Leeuwenhoek 1632–1723.

## Finding antique references

Finding antique references can be challenging, although the situation is improving as more libraries are being digitised.

Old books: Most of the antique books listed here can be found as free downloads in various formats on the Internet Archive. Sometimes it is necessary to search Google’s database. A reasonably fast internet connection is necessary for they are very large files.

Van Leeuwenhoek’s letters: The situation can be complicated. Most of his letters to the Royal Society were published, at least in part, in the Philosophical Transactions of the Royal Society, which can be searched on their online publishing archive (Phil Trans). However, the editors of 3 centuries ago edited most of the letters they published quite heavily, no matter who the author was. For some letters, only abstracts were included. Certain editors concentrated on publishing physics and mathematics communications, and biology and chemistry letters were avoided. When Van Leeuwenhoek realised that his letters were not appearing in the Philosophical Transactions, he started self-publishing collections of his work in the Dutch Language (or sometimes Latin). Those books will be listed as citations below, as necessary. They are worth checking as figures that have been lost from other versions are frequently included.

Unpublished Letters: Dobell (1932) listed 27 letters to the Royal Society that he said had never previously been published. All 27 can be found in volume 1 of the Collected Letters. Other letters were sent to different contacts ranging from the Mayor of Delft to the Librarian at the Vatican.

The Collected Letters (or “Alle de Brieven”): Anyone with an interest in the work of Antoni van Leeuwenhoek owes a special debt of gratitude to the editorial committees and editors of “Alle de Brieven” (Robertson et al 2016). They began work in 1931 and when it is complete, the series will contain all surviving letters from van Leeuwenhoek to his various correspondents, including the complete versions of the extracts published in the Royal Society’s “Philosophical Transactions”, those listed as “unpublished” (above) and in a volume edited by Van Rijnberk (1930). The letters (including those originally written in Latin) are presented in modern Dutch and English on facing pages, and include some of Van Leeuwenhoek’s own illustrations as well as other relevant pictures. Volume 1 appeared in 1939, and Volumes 18 and 19 should appear in 2023, when the series will be complete.

## References to Van Leeuwenhoek’s letters

In view of the confusion described above, references to the letters will be given here linked to Van Leeuwenhoek’s name and the year of the original publication to maintain the chronological sequence of the work, and with the reference to the Collected Letters. Volumes 1–15 are available free online as pdfs from DBNL. Volumes 16 and 17 are published by CRC Press. Volumes 18 and 19 are in press, and the necessary page numbers below were kindly provided by one of the editors, Douglas Anderson.

## References

[CR1] Académie des Sciences (2023) List of members during the creation of the Academie https://www.academie-sciences.fr/en/Liste-des-membres-depuis-la-creation-de-l-Academie-des-sciences/les-membres-du-passe-dont-le-nom-commence-par-l.html

[CR2] Adams G (1746) Micrographia Illustrata OR The knowledge of the microscope explained, 1st edn. George Adams, London, pp 1–6

[CR3] Anderson D (2014). Still going strong: Leeuwenhoek at eighty. Antonie Van Leeuwenhoek J Microbiol.

[CR4] Anderson D (2023a) Leeuwenhoek’s Cabinet of Wonders. https://lensonleeuwenhoek.net/content/leeuwenhoeks-cabinet-wonders Accessed 7 Mar 2023a

[CR5] Anderson (2023b) Lens on Leeuwenhoek, http://lensonleeuwenhoek.net/ Accessed 7 Mar 2023b

[CR6] Baker H (1742) The microscope made easy. London: R & J Dodsley

[CR7] BBC (2011) The hidden kingdom episode 1. https://www.bbc.co.uk/programmes/b00m425d Accessed 7 Mar 2023

[CR8] Birch T (1757) The history of the Royal Society of London for improving of naturalknowledge, from its first rise. 4: Royal Society, London

[CR9] Boitet R (1729) Beschrijving der stadt Delft. 2nd edn. Reinier Boitet, Delft

[CR10] Bolt M, Cocquyt T, Korey M (2018). Johannes Hudde and his flameworked microscope lenses. J Glass Stud.

[CR11] Bradley R (1721) The plague at Marseilles considered with remarks upon the plague in general, shewing its cause and nature of infection, with necessary precautions to prevent the spreading of that direful distemper. Bradley, London

[CR12] Clifton G (1996). Directory of british scientific instrument makers 1550–1851.

[CR13] Cocquyt T (2015). De identificatie van een zilveren microscoopje van Antoni van Leeuwenhoek (1632–1723). Studium.

[CR14] Cocquyt T (2022). Positioning Van Leeuwenhoek’s microscopes in 17^th^ century microscopic practice. FEMS Microbiol Lett.

[CR15] Cocquyt T, Zhou Z, Plomp J, van Eijck L (2021). Neutron tomography of Van Leeuwenhoek’s microscopes. Sci Adv.

[CR16] Davis I (2020). Antoni van Leeuwenhoek and measuring the invisible: the context of 16th and 17th century micrometry. Stud History Philosophy Sci Part A.

[CR17] Davis I (2022). Antoni van Leeuwenhoek: defining proportion in the microscopic realm during the 17^th^ century. FEMS Microbiol Lett.

[CR18] DBNL. Digitale Bibliotheek voor de Nederlandse Letteren, Den Haag: Royal Library. http://www.dbnl.org/auteurs/auteur.php?id=leeu027 Accessed 21 Mar 2023

[CR19] De Clerck P (1997). At the sign of the oriental lamp.

[CR87] De Kruif P (1926). Microbe hunters pocket books New York.

[CR20] Dobell C (1932). Antony van Leeuwenhoek and His “little animals”.

[CR21] FEMS (2014) FEMS Image Contest https://oupacademic.tumblr.com/post/103718754372/fems-image-contest. London and New York: Oxford University Pres. Accessed 7 Mar 2023

[CR22] Flores DP, MarzulloT C (2021). (2021) The construction of high-magnification homemade lenses for a simple microscope: an easy “DIY” tool for biological and interdisciplinary ecucation. Adv Physiol Educ.

[CR23] Folkes M (1723) Some account of Mr Leeuwenhoek’s curious microscopes lately presented to the Royal Society. Philosophical Trans Royal Society of London. 32:446–453

[CR24] Ford BJ (1981). The Van Leeuwenhoek specimans. Notes Rec R Soc Lond.

[CR25] Geesteranis J (1745) Inventory of the Estate of Maria van Leeuwenhoek. 2971. Delft City Archives, Delft

[CR26] Haaxman P (1875) Antoni van Leeuwenhoek. Leiden, Van Doesburgh

[CR27] Hooke R (1665) Micrographia or some physiological descriptions of minute bodies made by magnifying glasses with observations and inquiries thereupon. London: Royal Society

[CR28] Hooke R (1679) Lectiones Cutleriane or a collection of lectures made before the Royal Society on several occasions at Gresham College. Royal Society, London

[CR29] Internet Archive. The Internet Archive, San Francisco. https://archive.org Accessed 22 Mar 2023

[CR30] Mayall John (1886). Cantor lectures. The microscope. Lecture II. J Soc Arts.

[CR31] Jurin J (1722a) Letter 27 In: Rusnock A (ed) (1996) The Correspondence of James Jurin (1684–1750). Editions Rodophi, Amsterdam and Atlanta, GA 99–101

[CR32] Jurin J (1722b) Letter 41 IN: Rusnock A (ed) (1996) The Correspondence of James Jurin (1684–1750). Editions Rodophi, Amsterdam and Atlanta, GA 120–122

[CR33] Loncke H (2006). Lensje maken deel 4, of maak zelf een van Leeuwenhoek microscoop. Microwereld.

[CR34] Loncke H (2006a} Lensje maken, deel 2. Microwereld 44:6–15

[CR35] Mol JC (1924) (director and cinematographer) Antony van Leeuwenhoek. https://www.youtube.com/watch?v=sNSWwym57Ho Bureau van Wetenschappelijke Kinematografie

[CR36] Muffet G (1634) Insectorum sive minimorum animalium theatrum. T. Cotes, London

[CR37] Phillips JC (1747) Detail from Rees (1747)

[CR38] Phil Trans. Archive of All Online Editions 1665–1887. Philosophical Transactions of the Royal Society. Royal Society Publishing. http://rstl.royalsocietypublishing.org/content/by/year Accessed 21 Mar 2023

[CR39] Press Release 15 October 2019, RijksmuseumBoerhaave.nl/oog-in-oog-met-oude-koe

[CR40] Quint KD, Robertson LA (2019). Historical microbiology–using a Van Musschenbroek Microscope. FEMS Microbiol Lett.

[CR41] Rees A (1747) Catalogus van het vermaarde cabinet van vergrootglasen met zeer veel moeite, wen kosten in veele jaren ge¨ınventeert, gemaakt, en nagelaten door wylen den Heer Anthony van Leeuwenhoek. Reinier Boitet, Delft

[CR42] Robertson LA (2015). Historical microbiology, is it relevant in the 21st century?. FEMS Microbiol Lett.

[CR43] Robertson LA (2015). And then there were 12—distinguishing Van Leeuwenhoek microscopes from old or new copies. FEMS Microbiol Lett.

[CR44] Robertson LA (2015). Van Leeuwenhoek microscopes—where are they now?. FEMS Microbiol Lett.

[CR45] Robertson LA (2017). Lighting Van Leeuwenhoek’s samples. FEMS Microbiol Lett.

[CR46] Robertson LA (2019). Was Antoni van Leeuwenhoeck secretive?.

[CR47] Robertson LA (2022). The vanishing link between animalcules and disease before the 19th century. FEMS Microbiol Lett.

[CR48] Robertson L, Backer J, Biemans C, van Doorn J, Krab K, Reijnders W, Smit H, Willemsen P (2016) Antoni van Leeuwenhoek, Master of the Miniscule. Brill, Leiden & Boston 2016

[CR49] Robertson LA (2014) Through van Leeuwenhoek’s Eyes. https://oupacademic.tumblr.com/post/103718754372/fems-image-contest Accessed 7 Mar 2023

[CR50] Rusnock A (ed) (1996) The Correspondence of James Jurin (1684–1750). Letter 34. P. 110–111. Editions Rodophi, Amsterdam and Atlanta, GA

[CR51] Underwood EA (1977). Boerhaave’s men at Leyden and after.

[CR52] Van Delft D (2022) Onzichtbaar Leven Prometheus, Amsterdam

[CR53] Van Egmond (2016) The riddle of the ”green streaks” Micscape magazine 239: http://www.microscopy-uk.org.uk/mag/artfeb16/wimleeuwenhoek2.html

[CR54] Van Leeuwenhoek (1680) In: Birch, T (1757) 4:11, The History of the Royal Society of London for improving of Natural Knowledge. Royal Society, London

[CR55] Van Leeuwenhoek A (1723a) De globulis in sanguine & in vini fœcibus. Epistola posthuma domini Antonij a Leeuwenhoek, Societatis Regiæ Londinensis, dum viveret, Sodalis dignissimi, ad Jacobum Jurin, R. S. Secr Phil Trans Royal Soc 32: 433–434 10.1098/rstl.1722.0085

[CR56] Van Leeuwenhoek A (1723b) Ejusdem viri clarissimi ad eundem epistola posthuma De generatione animalium de palpitatione diaphragmatis. Phil Trans Royal Soc 32: 438–440 10.1098/rstl.1722.0088

[CR57] Van Leeuwenhoek A (1676a) Letter No 25 In: The Collected Letters of Antoni van Leeuwenhoek 2:43- 59 Pub Swets & Zeitlinger Ltd Amsterdam

[CR58] Van Leeuwenhoek A (1676b) Letter No 26 In: The Collected Letters of Antoni van Leeuwenhoek 2:61–161 Pub Swets & Zeitlinger Ltd Amsterdam

[CR59] Van Leeuwenhoek A (1677) Letter No 35 In: The Collected Letters of Antoni van Leeuwenhoek 2:277–299 Pub Swets & Zeitlinger Ltd Amsterdam

[CR60] Van Leeuwenhoek A (1679) Letter No 42 In: The Collected Letters of Antoni van Leeuwenhoek 2:409–423. Pub Swets & Zeitlinger Ltd Amsterdam

[CR61] Van Leeuwenhoek A (1687) Letter No 101 In: The Collected Letters of Antoni van Leeuwenhoek 6:311–345 Pub Swets & Zeitlinger Ltd Amsterdam

[CR62] Van Leeuwenhoek A (1689) Letter No 113 In: The Collected Letters of Antoni van Leeuwenhoek 8:67–117. Pub Swets & Zeitlinger Ltd Amsterdam\

[CR63] Van Leeuwenhoek A (1691) Letter No 115 In: The Collected Letters of Antoni van Leeuwenhoek 8:181–189 Pub Swets & Zeitlinger Ltd Amsterdam

[CR64] Van Leeuwenhoek A (1693) Aan Haar Majesteit Maria, Koninginne van Groot Brittanjen. Dedication in Derde Vervolgder Brieven, Pub Henrik van Krooneveld, Delft

[CR65] van Leeuwenhoek A (1694) Vierde Vervolg Der Brieven. HA Krooneveld, Delft

[CR66] Van Leeuwenhoek A (1695) *Arcana Naturae Detecta*. Delphis Batavorum: H. A. Krooneveld Delft

[CR67] Van Leeuwenhoek A (1696) Vijfde Vervolg der Brieven, HA Krooneveld, Delft

[CR68] Van Leeuwenhoek A (1699) Letter No 200 In: The Collected Letters of Antoni van Leeuwenhoek 12:293–309 Pub Swets & Zeitlinger Ltd Amsterdam

[CR69] Van Leeuwenhoek A (1703) Letter No 240 In: The Collected Letters of Antoni van Leeuwenhoek 12:243–267 Pub Swets & Zeitlinger Ltd Amsterdam

[CR70] Van Leeuwenhoek A (1710) Letter No 282 In: The Collected Letters of Antoni van Leeuwenhoek 16:191–209 Pub Taylor & Francis London

[CR71] Van Leeuwenhoek A (1711) Letter No 287 In: The Collected Letters of Antoni van Leeuwenhoek 16:265–275 Pub Pub Taylor & Francis London

[CR72] Van Leeuwenhoek A (1713) Letter No 299 In: The Collected Letters of Antoni van Leeuwenhoek 17: 75–85 Pub Taylor & Francis London

[CR73] Van Leeuwenhoek A (1714) Letter No 307 In: The Collected Letters of Antoni van Leeuwenhoek 17:195–219 Pub Pub Taylor & Francis London

[CR74] Van Leeuwenhoek A (1716) Letter No 324 In: The Collected Letters of Antoni van Leeuwenhoek 18:17–27. In Press

[CR75] Von Uffenbach ZC (1754) Merkwürdige Reisen durch Niedersachsen, Holland und Engelland Band 3. Gaum, Ulm

[CR76] van Loon G (1731) Beschryving Der Nederlandsche Historipenningen III. van Lom C, Gosse P, Alberts R, de Hondt P, ’s Gravenhage

[CR77] Van Seters WH (1951). Antoni van Leeuwenhoek in Amsterdam. Notes Rec R Soc Lond.

[CR78] Van Seters WH (1968). Van Leeuwenhoeks tweede huwelijk. Ned Tijdschr Geneeskd.

[CR79] Van Seters WH, Palm LC, Snelders HAM (1982). Can Antoni van Leeuwenhoek have attended school at Warmond?. Antoni van Leeuwenhoek, 1632–1723: studies on the life and work of the Delft scientist.

[CR80] Van Zuylen J (1981). The microscopes of Antoni van Leeuwenhoek. J Microsc.

[CR81] Van Rijnberk G (editor) (1930) Fourteen hitherto totally unpublished papers of Antoni van Leeuwenhoek of the years 1674–1678. Opuscula selecta Neerlandicorum de Arte Medica 9

[CR82] Verkolje J (1686) Mezzotint Portrait of Antoni van Leeuwenhoek. Leiden: Museum Boerhaave

[CR83] Visualizing the Unknown (2021) https://visualizingtheunknown.com/ Accessed 7 March 2023

[CR84] Wassink, Jos. (2018) Neutron beams reveal Van Leeuwenhoek’s secret. Delta Journalistic Platform, TU Delft 26 March 2018. https://www.delta.tudelft.nl/article/neutron-beams-reveal-van-leeuwenhoeks-secret.

[CR85] William S (1702) Pharmacopoeia Londinensis OR The New London Dispensatory J.Dawks, London

[CR86] Zuidervaart, HJ, Rijks M. “Most rare workmen”: Optical practitioners in Early 17th century Delft”. The British Journal for the History of Science DOI: 10.1017/S00070877777414000181 pp 1–33. 10.1017/S0007087414000181 25833798

